# Limited Genetic Diversity Detected in Middle East Respiratory Syndrome-Related Coronavirus Variants Circulating in Dromedary Camels in Jordan

**DOI:** 10.3390/v13040592

**Published:** 2021-03-31

**Authors:** Stephanie N. Seifert, Jonathan E. Schulz, Stacy Ricklefs, Michael Letko, Elangeni Yabba, Zaidoun S. Hijazeen, Peter Holloway, Bilal Al-Omari, Hani A. Talafha, Markos Tibbo, Danielle R. Adney, Javier Guitian, Nadim Amarin, Juergen A. Richt, Chester McDowell, John Steel, Ehab A. Abu-Basha, Ahmad M. Al-Majali, Neeltje van Doremalen, Vincent J. Munster

**Affiliations:** 1 Virus Ecology Section, Laboratory of Virology, National Institutes of Allergy and Infectious Diseases, National Institutes of Health, Rocky Mountain Laboratories, Hamilton, MT 59840, USA; jonathan.schulz@nih.gov (J.E.S.); michael.letko@wsu.edu (M.L.); elangeniyabba96@gmail.com (E.Y.); danielle.adney@nih.gov (D.R.A.); neeltje.vandoremalen@nih.gov (N.v.D.); vincent.munster@nih.gov (V.J.M.); 2 Paul G. Allen School for Global Health, Washington State University, Pullman, WA 99164, USA; 3 Rocky Mountain Labs Genomics Unit, National Institutes of Allergy and Infectious Diseases, National Institutes of Health, Rocky Mountain Laboratories, Hamilton, MT 59840, USA; sricklefs@niaid.nih.gov; 4 National Consultant, Food and Agriculture Organization of the United Nations, Amman 11194, Jordan; kalanzi80@yahoo.com; 5 Economics and Public Health Group, Royal Veterinary College, Hatfield AL9 7TA, UK; pholloway3@rvc.ac.uk (P.H.); jguitian@rvc.ac.uk (J.G.); 6 Department of Veterinary Medicine, Jordan University of Science and Technology, Irbid 22110, Jordan; bilal@just.edu.jo (B.A.-O.); hanit@just.edu.jo (H.A.T.); abubasha@just.edu.jo (E.A.A.-B.); almajali@just.edu.jo (A.M.A.-M.); 7 Subregional Office for the Gulf Cooperation Council States and Yemen, Food and Agriculture Organization of the United Nations, Abu Dhabi 62072, United Arab Emirates; markos.tibbo@fao.org; 8 Technical and Marketing Department, United Animal Health, Sheridan, IN 46069, USA; nadim.amarin@unitedanh.com; 9 Department of Diagnostic Medicine and Pathobiology, Kansas State University, Manhattan, KS 66506, USA; jricht@vet.k-state.edu (J.A.R.); cdmcdow@vet.k-state.edu (C.M.); 10 Department of Microbiology and Immunology, Emory University, Atlanta, GA 30322, USA; pdx1@cdc.gov; 11 Influenza Division, Centers for Disease Control, Atlanta, GA 30329, USA

**Keywords:** viral genomics, MERS-CoV, zoonoses, coronaviruses, phylogenomics, population genomics

## Abstract

Middle East respiratory syndrome-related coronavirus (MERS-CoV) is a persistent zoonotic pathogen with frequent spillover from dromedary camels to humans in the Arabian Peninsula, resulting in limited outbreaks of MERS with a high case-fatality rate. Full genome sequence data from camel-derived MERS-CoV variants show diverse lineages circulating in domestic camels with frequent recombination. More than 90% of the available full MERS-CoV genome sequences derived from camels are from just two countries, the Kingdom of Saudi Arabia (KSA) and United Arab Emirates (UAE). In this study, we employ a novel method to amplify and sequence the partial MERS-CoV genome with high sensitivity from nasal swabs of infected camels. We recovered more than 99% of the MERS-CoV genome from field-collected samples with greater than 500 TCID_50_ equivalent per nasal swab from camel herds sampled in Jordan in May 2016. Our subsequent analyses of 14 camel-derived MERS-CoV genomes show a striking lack of genetic diversity circulating in Jordan camels relative to MERS-CoV genome sequences derived from large camel markets in KSA and UAE. The low genetic diversity detected in Jordan camels during our study is consistent with a lack of endemic circulation in these camel herds and reflective of data from MERS outbreaks in humans dominated by nosocomial transmission following a single introduction as reported during the 2015 MERS outbreak in South Korea. Our data suggest transmission of MERS-CoV among two camel herds in Jordan in 2016 following a single introduction event.

## 1. Introduction

Middle East respiratory syndrome coronavirus (MERS-CoV) is a zoonotic betacoronavirus with frequent spillover from dromedary camels into humans, primarily in the Eastern Mediterranean Region. Human-to-human transmission is limited with the exception of hospital settings where nosocomial transmission leads to outbreaks of MERS characterized by severe respiratory distress and a case-fatality rate of over 30% in confirmed cases. The majority of confirmed MERS cases have occurred in the Kingdom of Saudi Arabia (KSA) [1] where large camel markets have been shown to support endemic transmission of diverse lineages of MERS-CoV among camels, including individuals infected with multiple MERS-CoV variants simultaneously [2]. Analysis of full MERS-CoV genome sequences sampled from large camel markets in the United Arab Emirates (UAE) revealed a similar pattern in co-housed camels shown to be infected with MERS-CoV from diverse genetic lineages [3]. Phylodynamic analyses supports that the evolution of MERS-CoV, including extensive recombination, occurs primarily in camels rather than in humans [4], suggesting that maintenance of MERS-CoV populations in camels is an important component of generating viral diversity. Availability of additional camel-derived MERS-CoV genomes will help to reveal further information about the diversity and epidemiology of MERS-CoV in its reservoir, the dromedary camel. 

In this study, we describe a long-range polymerase chain reaction (LRPCR) assay to amplify the MERS-CoV genome from field-collected samples for next generation sequencing (NGS). We demonstrate the utility and sensitivity of this assay by sequencing the genome of MERS-CoV from nasal swabs collected from infected camels in Jordan in May 2016 for which partial spike S2 sequences had been recovered for 16 of 28 camel swabs that had tested positive for MERS-CoV by RT-PCR [5]. We then analyze the genomic sequences from these camels to better understand the population dynamics of MERS-CoV in Jordan where there have been sporadic human MERS outbreaks since 2012 [6,7]; however, as of yet, no camel-derived full-length MERS-CoV genome sequences have been published from Jordan. 

## 2. Materials and Methods

Nasal swabs were collected from camels as described by van Doremalen et al., 2017 [5]. 

### 2.1. Amplification of MERS-CoV Genome

**Figure 1:**
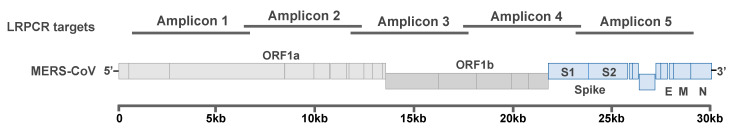
Location of five, approximately 6 kb, overlapping LRPCR amplicons spanning 99% of the MERS-CoV genome. Nucleotide positions relative to the 2012 EMC variant MERS-CoV reference genome, NCBI Accession #JX869059, are listed under each amplicon name for the first and second round (A and B, respectively) of the semi-nested LRPCR.

We designed a semi-nested LRPCR assay to produce 5 overlapping amplicons, approximately 6-kilobase pairs (kbp) each in length, spanning approximately 99% of the MERS-CoV genome, including all protein-coding sequences (Figure 1). This method has been applied to sequencing the Ebola virus [8] and henipavirus [9] genomes from complex samples and is compatible with a variety of next generation sequencing platforms, including the field-portable Oxford MinION [8,9]. Primers were designed targeting conserved regions in order to amplify across all presently described MERS-CoV lineages (Table 1). Following cDNA synthesis as described in Seifert et al. [8], 5 μL of template cDNA was added to a reaction mix for each amplicon containing 10 μL of CloneAMP HiFi PCR PreMix (Takara Bio USA, Inc., Mountain View, CA, USA), 0.4 μM of each primer, and H_2_O to 25 μL. The reaction mix was then thermal cycled 35 times at 98 °C for 10 s, 56 °C for 15 s, and 72 °C for 30 s. The process was repeated, with 2 μL of the first-round LRPCR used as template in a second round of LRPCR before visualizing by gel electrophoresis.

### 2.2. Library Preparation and Sequencing

LRPCR products were purified with AMPure XP beads as previously described in Seifert et al. [8], pooled at equal molar ratios, prepped for NGS using the TruSeq Nano DNA kit, and paired-end sequencing was completed on the MiSeq platform (Illumina, Inc., San Diego, CA, USA). The BBTools bioinformatics suite (https://sourceforge.net/projects/bbmap/, accessed on 1 February 2021) was used to trim adapter and indexing primer sequences, filter low-quality bases (<Q30), and remove duplicate reads before mapping to a reference MERS-CoV genome sequence (JX869059.2) using BBMap v 1.0. The average depth of coverage was greater than 50× for all samples. Variant calling was completed using FreeBayes v 1.1.0-50-g61527c5 using the following parameters: p, 1 pooled-continuous; min-alternate-fraction, 0.3; min-alternate-count, 10 [10]. Sequences with greater than or equal to 80% of the MERS-COV genome recovered were annotated and deposited in GenBank under the accession numbers MW086527–MW086540.

### 2.3. Phylogenomic Analyses

All MERS-CoV genome sequences (≥20,000 bp in length, from 2012 through to March 2021) available on GenBank were downloaded and curated to remove sequences representing serial sampling studies, sequences lacking metadata, and sequences from cell-culture adaptation studies. Fourteen full camel-derived MERS-CoV genome sequences and 6 partial MERS-CoV genome sequences (ranging from 5–18 kb) from this study were then aligned with the remaining 464 MERS-CoV genomes, and the maximum likelihood phylogenetic tree was inferred with 1000 UltraFast bootstrap replicates implemented in IQ-TREE v 2.1.2 [11], with integrated ModelFinder used to determine the best-fit substitution model, with GTR +F+R3 chosen with the Bayesian Information Criterion [12]. 

As recombination may confound phylogenetic signal, recombination detection program 4 (RDP4 [13]) was used to investigate the effect of recombination on the topology of the MERS-CoV phylogenomic tree pertaining to the placement of the Jordan camel-derived variants sequenced for this study. Recombination analyses were performed in RDP4 using the 3seq, bootscan, genecov, maxchi, lard, and chimaera detection methods, and we considered recombination events to be reliable when detected by two or more methods. The Jordan camel-derived MERS-CoV variants sequenced for this study were not flagged as recombinants, nor were the KSA variants sharing a most recent common ancestor with the Jordan sequences. We therefore removed recombinant sequences from the alignment and reconstructed a second maximum likelihood tree with the remaining 425 MERS-CoV genomes. In each phylogenetic tree, the Jordan camel-derived sequences most closely clustered with contemporaneous lineage B.5 MERS-CoV variants identified in KSA. To infer a time-resolved phylogenetic tree, lineage A and C MERS-CoV variants were removed from the alignment containing 425 MERS-CoV genomes with recombinant sequences stripped, and a timetree was inferred with the remaining 403 MERS-CoV genomes using TreeTime v 0.7.6 [14] with a coalescent skyline and fixed clock rate of 9.57 × 10^−4^ subs/site/year reflecting the evolutionary rate estimated by Dudas et al. [4].

## 3. Results

**Figure 2:**
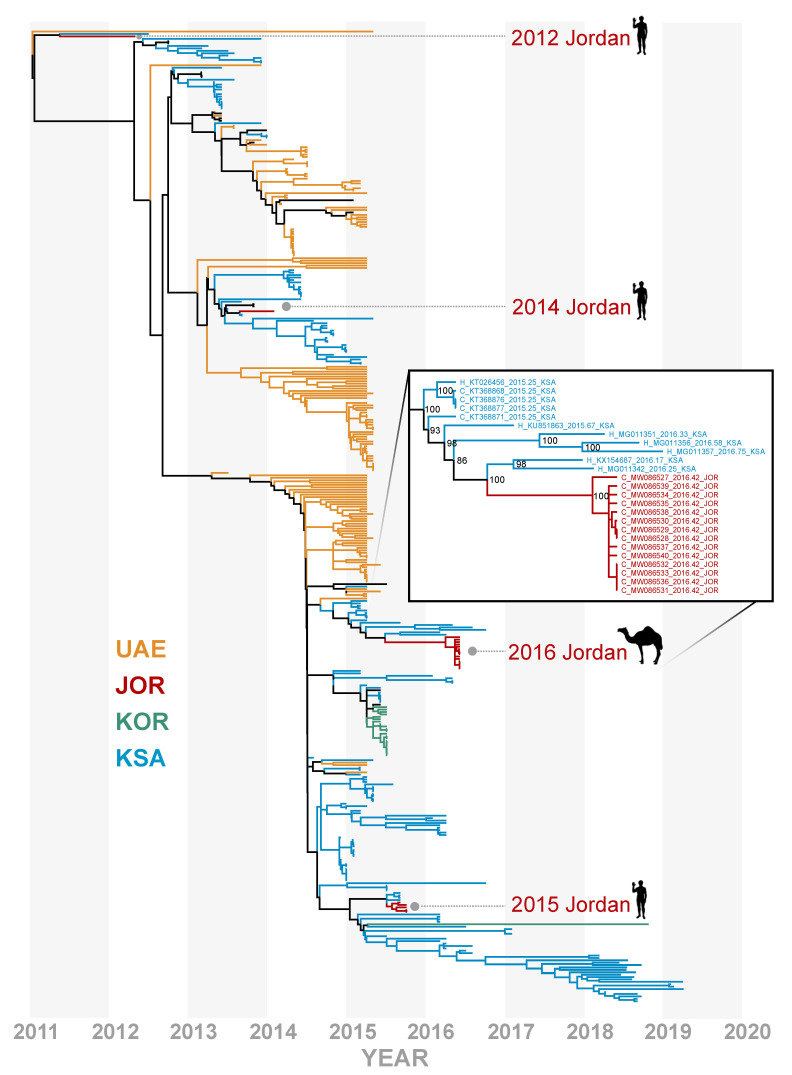
Time-resolved phylogenomic tree inferred from full genome sequences for 403 lineage B MERS-CoV genomes with recombinant sequences removed. Variants from United Arab Emirates (UAE) in mustard yellow, South Korea (KOR) in green, Saudi Arabia (KSA) in blue, and Jordan (JOR) in dark red. All MERS-CoV sequences originating from JOR through time are highlighted. Odd years are shown in dark-gray bands. Fourteen camel-derived MERS-CoV genomes from this study are highlighted with an inset showing bootstrap support at nodes in context of the most phylogenetically similar variants.

All 5 amplicons were recovered for samples with greater than 500 equivalent (eq) 50% tissue culture infectious dose (TCID_50_)/swab as reported in [5], with variable success for samples with less than 500 TCID_50_eq/swab (Figure S1), resulting in 14 camel-derived MERS-CoV sequences with more than 80% of the genome recovered and 6 camel-derived MERS-CoV sequences with less than 80% of the genome recovered. The Jordan camel-derived MERS-CoV sequences clustered most closely with lineage B.5 MERS-CoV variants sampled in Riyadh, KSA in early 2016 (Figure 2, Figures S2 and S3). There have been few studies to characterize whether diversification in MERS-CoV correlates to functional variation in pathogenesis, environmental stability, or transmission efficiency. However, a deletion in ORF4b has been linked to a decrease in interferon antagonism in MERS-CoV variants derived from camels in Africa [15]. 

Our full-genome analysis contradicts the earlier placement of these sequences with lineage B.1 which had low support in a maximum likelihood tree inferred from the partial S2 subunit with few lineages represented [5]. Furthermore, these camel-derived MERS-CoV variants do not cluster with the human cases reported in Jordan in 2015, suggesting that these variants represent a separate introduction of MERS-CoV. While full genome analysis can improve phylogenetic inference, we caution that lineage assignment with MERS-CoV is complicated by a history of extensive recombination and may not be phenotypically informative. The full genome sequences for these samples reveal that the May 2016 camel-derived MERS-CoV variants lack the unique deletion variants identified in the 2015 MERS outbreak in Jordan [7]. 

**Figure 3:**
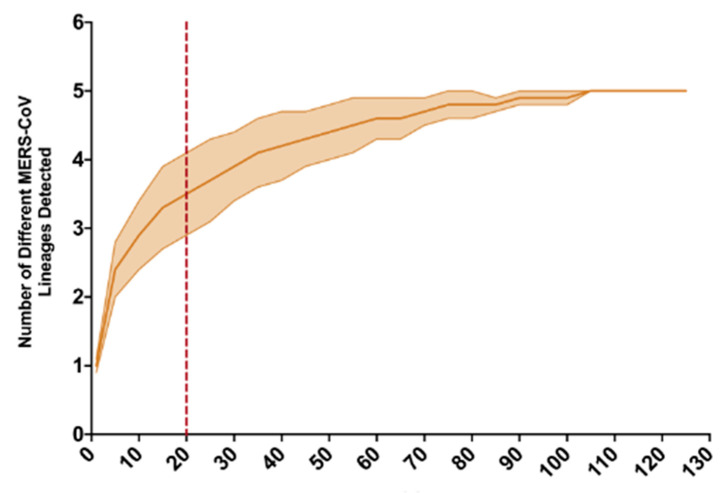
Rarefaction analysis of lineage assignment for 129 camel-derived full MERS-CoV genome sequences from a large camel market in UAE, as reported in Yusof et al., 2018. The envelope reflects variance, and the red dashed line at 20 samples reflects the number of MERS-CoV sequences recovered in this study, all of which belong to lineage B.5.

The Jordan camel-derived MERS-CoV genomes showed little genetic variation when compared to a study of a large camel market in UAE, where the recovered MERS-CoV genomes represented multiple MERS-CoV lineages in a single camel pen [3]. Average evolutionary divergence over sequence pairs was estimated in MEGA7 [16] within each of four groups: Jordan camel-derived MERS-CoV genome sequences from this study, human-derived MERS-CoV sequences during an outbreak following a single introduction event in South Korea, and large camel markets in UAE and KSA. Diversity within the Jordan camel-derived MERS-CoV genomes (d = 1.04 × 10^−4^) was more reflective of the limited diversity found during a nosocomial MERS outbreak following the introduction of a single variant in South Korea in 2015 (d = 8.91 × 10^−5^) than the UAE camel market study (d = 1.04 × 10^−3^) or the Jeddah camel market in KSA (d = 1.48 × 10^−3^). As more individual camels were sampled in the UAE study, we conducted a rarefaction analysis and determined that a sample size of 20 genomes from the UAE camel market study would yield an average of 3.5 MERS-CoV lineages (σ^2^ = 0.6) detected, whereas only one lineage was represented in the Jordan camel-derived genomes (Figure 3). Sabir et al. detected MERS-CoV variants from three lineages, with partial genome sequences recovered for just 18 MERS-CoV positive samples collected from a large camel market in Jeddah, KSA in December, 2014 [2].

Only a single MERS-CoV genome could be recovered from the Azraq camel herd, as the majority of samples from this herd had less than 500 TCID_50_eq/swab, whereas 13 full or partial genomes were recovered from the Ramtha herd [5]. The higher Ct values for the Azraq herd are consistent with sampling at the tail-end of the outbreak in that population. In a follow-up sampling mission, nasal swabs from 26 camels were collected from the Ramtha herd in October 2017, and none of the samples were positive for MERS-CoV RNA by RT-qPCR, compared to 17 of 22 camels testing positive for MERS-CoV RNA in May 2016 as reported in van Doremalen et al. 2017 [5]. 

## 4. Discussion

There have been comparatively few confirmed MERS cases in Jordan relative to neighboring KSA despite a similar reliance on camels as livestock. An outbreak of MERS in Jordan reported in 2015 led to a follow-up study in 2016 to determine the prevalence of MERS-CoV in Jordan camel herds, revealing a high seroprevalence against MERS-CoV and a high proportion of nasal swab samples which tested positive by qRTPCR for the presence of MERS-CoV RNA [5]. However, full viral genomes could not be recovered from the samples at the time, and the partial S2 sequences recovered were not phylogenetically informative for delineating whether the 2016 MERS-CoV variants detected in Jordan camels were closely related to the sequences from the 2015 MERS outbreak. In this study, we reliably recovered nearly complete MERS-CoV genomes from nasal swabs collected from camels, with viral titers as low as 500 TCID_50_eq/swab, using a novel LRPCR assay (Figure S1). Our phylogenetic analyses support the hypothesis that the MERS-CoV variants sequenced from humans in Jordan in 2015 stemmed from a separate introduction to Jordan than the MERS-CoV variants circulating in camels in 2016 (Figure 2). Our analyses show a striking lack of genetic diversity in the MERS-CoV variants circulating in Jordan camels in 2016, with a subsequent sampling mission in 2017 yielding no MERS-CoV RNA detected in the Ramtha herd. Taken together, we hypothesize that MERS-CoV is not maintained in the smaller camel herds of Jordan, but instead, is frequently introduced to the region through camel trade or travel. 

While the majority of confirmed MERS cases have occurred in KSA and other countries in the Arabian Peninsula, MERS-CoV genomic material has also been recovered from camels throughout Africa [15,17,18]. MERS-CoV genome sequences described by Chu et al. revealed discrete regional patterns of genetic diversity in Africa [15], suggesting structured populations of MERS-CoV circulating in African camel herds. A recent study reported a high incidence of MERS-CoV RNA detected in camels from eastern Africa on import vessels destined for markets in Saudi Arabia, likely providing an opportunity for mixing between previously geographically isolated lineages [19]. Consistent with these observations, studies have shown extensive recombination between MERS-CoV lineages in the large camel markets of KSA and UAE [2,3]. In contrast, camels in Jordan are primarily maintained in smaller herds, with fewer, smaller camel markets in contrast to the large camel markets found in KSA and UAE. We hypothesize that camel management practices, including import and export and herd size, influence the diversity and maintenance of MERS-CoV in the reservoir; however, longitudinal sampling of camel herds representative of the diversity of camel management practices on a broad scale is needed to further support this hypothesis.

## Figures and Tables

**Table 1:** Primer sequences for each of five, approximately 6 kb amplicons, spanning the MERS-CoV genome. Each amplicon is the product of a semi-nested LRPCR.

Amplicon	Forward Primer Sequence (5′–3′)	Reverse Primer Sequence (5′–3′)	Round of LRPCR
1A	AACGAACTTAAATAAAAGCCCTGTTGTTT	GGGCATCTTCAAACATAACATCACTT	Round 1
2A	CACTTTCACTGCTACCACTGCTGTA	CGCGAAGTTTATTTGAAGCACA	
3A	CGCCTATGAGAAGGATAAGGCAGT	GATGCAGACGTTAATTCAAAGCCAT	
4A	GCCAGTTGGTGTTGTAGACACTGA	TTGCTAGGGTAATAACCAACATGCAT	
5A	GCTCGTGATCTTATTTGTGCTCAA	GCAGAGGTGACAGTCTTTAACATTCTCT	
1B	AACGAACTTAAATAAAAGCCCTGTTGTTT	ATACTTAAATCAACAGCAGCAGTGCAA	Round 2
2B	CACTTTCACTGCTACCACTGCTGTA	CTAAGAGGTATACAACCATTCCTAGCGTT	
3B	CGCCTATGAGAAGGATAAGGCAGT	ACGAGGTGCTTAAACTGTTCACCT	
4B	GCCAGTTGGTGTTGTAGACACTGA	CTGCTATGCTGCCAAGCAAA	
5B	GCTCGTGATCTTATTTGTGCTCAA	TGTGCAAGAGTGGACAAGCGAT	

## Data Availability

MERS-CoV full and partial genome sequence data are available through GenBank accession numbers MW086527–MW086540. Sequence data were deposited with relevant sampling date, host source, and geographic region.

## Supplementary Materials

The following are available online at https://www.mdpi.com/article/10.3390/v13040592/s1. Figure S1. Recovery of LRPCR amplicons across the MERS-CoV genome relative to the TCID50eq/swab reported for the UpE gene in van Doremalen et al. [5]; Figure S2. Phylogenetic tree of 484 MERS-CoV genomes estimated by maximum likelihood method in IQ-TREE v. 2.1.2, showing the consensus of 1000 ultrafast bootstrap replicates and rooted at the midpoint; Figure S3. Phylogenetic tree of 425 MERS-CoV genomes, with recombinant sequences stripped, estimated by maximum likelihood method in IQ-TREE v. 2.1.2, showing the consensus of 1000 ultrafast bootstrap replicates and rooted at the midpoint.
