# Supplementary material for: Limited Genetic Diversity Detected in Middle East Respiratory Syndrome-Related Coronavirus Variants Circulating in Dromedary Camels in Jordan
Source: Viruses. 2021 Mar 31;13(4):592. doi: 10.3390/v13040592 (PMC8067259; doi:10.3390/v13040592)
Supplement: Supplementary file 1 [file viruses-13-00592-s001.pdf]

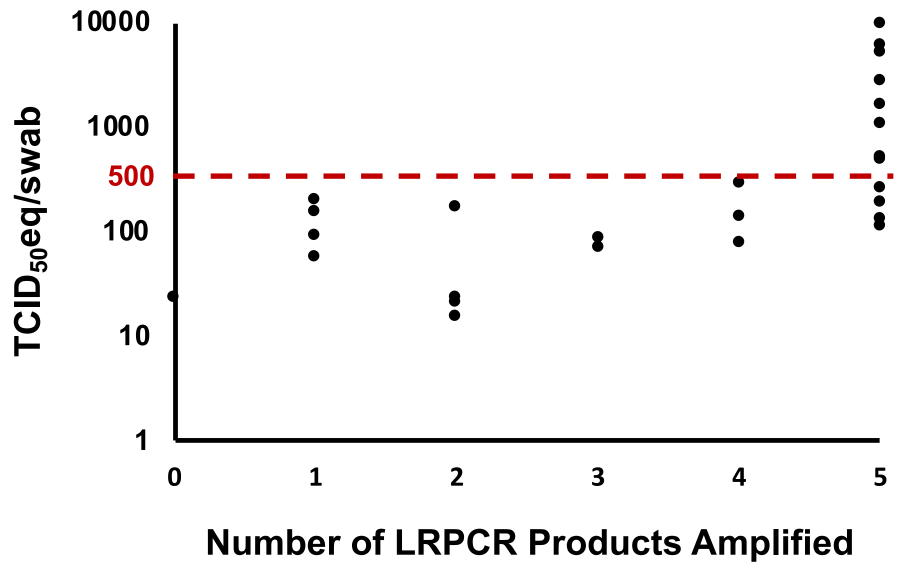


**Figure S1.** Sensitivity of the LRPCR assay when amplifying the MERS-CoV genome field collected camel nasal swabs. $TCID_{50}$ equivalent per swab for each sample is shown on the y-axis as determined by qRTPCR in van Doremalen et al. 2017 [5]. Number of LRPCR amplicons successfully recovered for each sample on the x-axis with 5 amplicons indicating recovery of more than 99% of the genome.

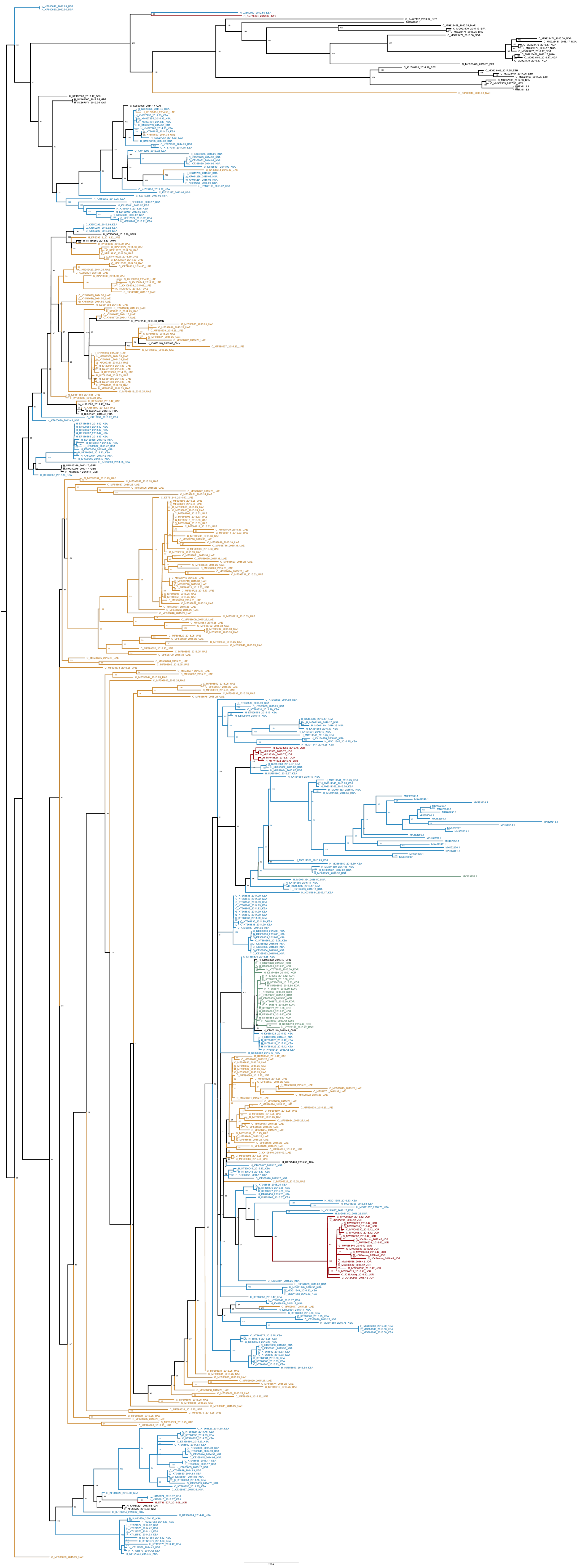

**Figure S2.** Maximum likelihood estimation of the phylogenetic relationships among 484 MERS-CoV genomes inferred with IQ-TREE and showing support at the nodes for 1000 bootstrap replicates.

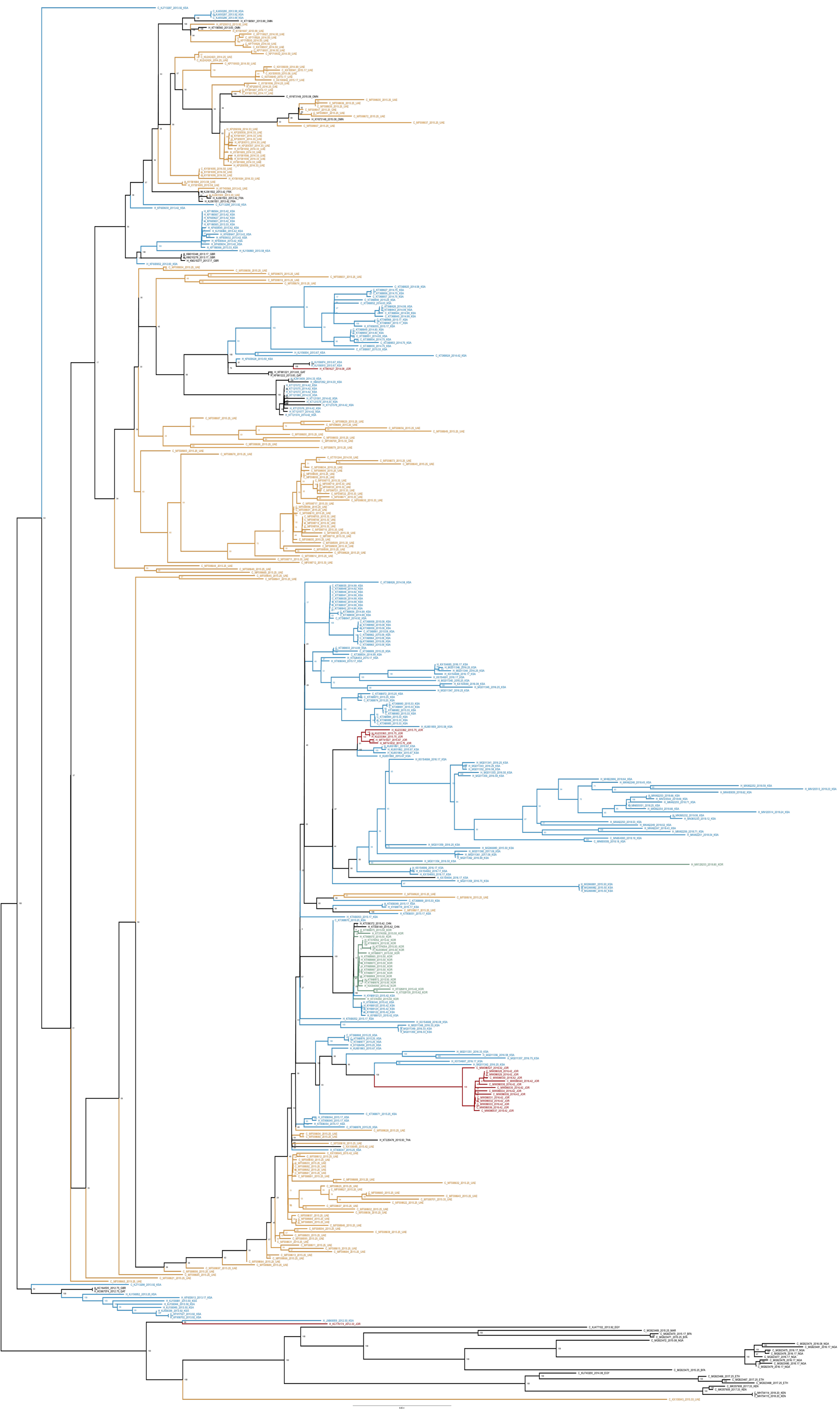

**Figure S3.** Maximum likelihood estimation of the phylogenetic relationships among 425 MERS-CoV genomes with recombinant sequences stripped. ML tree inferred with IQ-TREE and showing support at the nodes for 1000 bootstrap replicates.
